# Reactogenicity and Immunogenicity of the Pfizer and AstraZeneca COVID-19 Vaccines

**DOI:** 10.3389/fimmu.2021.794642

**Published:** 2021-12-01

**Authors:** Waleed H. Mahallawi, Walaa A. Mumena

**Affiliations:** ^1^ Medical Laboratory Technology Department, College of Applied Medical Sciences, Taibah University, Madinah, Saudi Arabia; ^2^ Clinical Nutrition Department, College of Applied Medical Sciences, Taibah University, Madinah, Saudi Arabia

**Keywords:** reactogenicity, immunogenicity, Pfizer, AstraZeneca, vaccines, Saudi Arabia

## Abstract

**Background:**

The relationships of the coronavirus disease 2019 (COVID-19) vaccination with reactogenicity and the humoral immune response are important to study. The current study aimed to assess the reactogenicity and immunogenicity of the Pfizer and AstraZeneca COVID-19 vaccines among adults in Madinah, Saudi Arabia.

**Methods:**

A cross-sectional study, including 365 randomly selected adult Pfizer or AstraZeneca vaccine recipients who received a homologous prime-boost vaccination between February 1^st^ and June 30^th^, 2021. Data of height and weight were collected to assess the weight status of percipients. An evaluation of seropositivity for anti-severe acute respiratory syndrome coronavirus 2 (SARS-CoV-2) antibodies was assessed using enzyme-linked immunosorbent assay (ELISA).

**Results:**

Among the participants, 69% (n = 250) reported at least one vaccine-related symptom. Pain at the injection site was the most frequently reported vaccine-related symptom. The mean total score for vaccine-related symptoms was significantly higher among participants who received the AstraZeneca vaccine, women, and participants with no previous COVID-19 infection (*p* < 0.05). Spike-specific IgG antibodies were detected in 98.9% of participants after the receipt of two vaccine doses, including 99.5% of Pfizer vaccine recipients and 98.3% of AstraZeneca vaccine recipients. Significantly, higher proportions of participants in the <35-year age group developed a humoral immune response after the first vaccine dose compared with the participants in other age groups.

**Conclusion:**

Participants who received the Pfizer COVID-19 vaccine reported fewer vaccine-related complications compared with those who received the AstraZeneca COVID-19 vaccine, but no serious side effects were reported in response to either vaccine. Health status and age were factors that may influence COVID-19 vaccine effectiveness for the generation of antibodies against the SARS-CoV-2 spike protein.

## Introduction

Coronavirus disease 2019 (COVID-19) has rapidly become a leading cause of death and both short- and long-term morbidity among individuals older than 45 years (1, 2), representing an extraordinary problem for healthcare organizations and causing global economic concerns and extended lockdowns (3).

Although COVID-19 vaccine efficacy and safety have been reported in recent studies (4–6), the current situation in Saudi Arabia has not been reported. Commonly, individuals with any history of prior COVID-19 infection, for whom vaccination is presently recommended (https://www.cdc.gov/coronavirus/2019-ncov/vaccines/faq.html, accessed on 11 October 2021), were omitted from vaccine-related clinical trials. Although previous infection with the severe acute respiratory syndrome coronavirus 2 (SARS-CoV-2) virus is thought to provokes a natural immunity that is durable for at least 6 months (7), whether prior SARS-CoV-2 infection is associated with vaccination side effects has not been determined (8).

Vaccines are desirable for controlling the spread of SARS-CoV-2 infections. COVID-19 vaccination, combined with other infection transmission prevention approaches, is necessary to prevent SARS-CoV-2 infections from spreading in the community, especially among those with immune disorders and health care workers who are fighting the disease on the front lines (9). Among the currently approved COVID-19 vaccines, the AstraZeneca vaccine is an adenovirus-based vector vaccine, and the Pfizer/BioNTech and Moderna vaccines are mRNA vaccines, all of which have been used in several settings, including Saudi Arabia (https://www.moh.gov.sa/en/Ministry/HotTopics/Pages/COVID-19-Vaccine.aspx, accessed on 11 October 2021). The AstraZeneca and Pfizer/BioNTech vaccines have demonstrated an immunogenic nature accompanied by significant effectiveness for preventing COVID-19 disease (4, 10). The mRNA-based Pfizer/BioNTech vaccine was the first vaccine to receive approval for use in Saudi Arabia in mid-December 2020, and the adenovirus-vectored AstraZeneca vaccine was the second vaccine to be approved in February 2021 (11).

Although these vaccines efficiently decrease the severity of the SARS-CoV-2 infection (12, 13), preliminary investigations of reactogenicity showed that transitory local responses were common and systemic episodes were rare following vaccination (14). One recent national study examined the reactogenicity of the AstraZeneca vaccine by collecting data from participants over the phone (15). However, dual comparative investigations of reactogenicity and immunogenicity between approved vaccines in Saudi Arabia have not been performed.

In the current study, we aimed to assess the reactogenicity and immunogenicity among adults in response to receiving both doses of the Pfizer and AstraZeneca vaccines and correlate the reactogenicity profiles and induction of humoral immunity with the characteristics of the participants.

## Materials and Methods

### Study Design and Population

This cross-sectional study recruited adult participants aged 18 years or older who received both doses of either the Pfizer or AstraZeneca COVID-19 vaccine and visited the vaccine center at Taibah University between February 1 and June 30, 2021. In accordance with recommendations, the time between the first and second Pfizer vaccinations was 3 weeks, whereas 12 weeks separated the administration of the AstraZeneca doses. The study was advertised at the vaccine center, and banners were placed in the waiting rooms. Initially, data were collected from 952 individuals who agreed to participate *via* an online survey. Participants were invited to an in-person visit, at which missing data were obtained, and a blood sample was collected. Individuals who reported recent contact with COVID-19–infected individuals were excluded. Written informed consent was obtained from each participant before any personal data were collected. An electronic survey was sent to the vaccine recipients one week following receipt of the first vaccine dose to collect data regarding sociodemographics, anthropometrics, and reactogenicity and invite them to participate in the collection of a blood sample. The reactogenicity data described in the current study involved the self-reported local and systemic symptoms experienced during the 7 days following the administration of the first vaccine dose. Ethical approval for this study was obtained from the ethical review board of the College of Applied Medical Sciences, Taibah University (2021/96/117/MLT).

### Data Collection

The online survey collected sociodemographic data, including age, sex, nationality, education level, and career. Data concerning blood type and medical history (previous infection with COVID-19) were also self-reported. According to medical history and self-reported health status, the responses were grouped into 3 categories: 1) healthy; 2) immune-related diseases (including type 1 diabetes, rheumatism, hypothyroidism, and Crohn’s disease); and 3) chronic diseases (including type 2 diabetes, hypertension, and cardiovascular disease). All self-reported data were collected using an online survey, and items were validated/completed during the in-person visit.

### Anthropometrics

Anthropometric measurements (height and weight) were collected *via* the online survey. Height and weight were used to calculate the body mass index (BMI) to determine the weight status of participants aged >18 years, using the World Health Organization cutoffs (underweight= BMI <18.5 kg/m^2^; healthy weight= BMI between 18.5 to <25.0 kg/m^2^; overweight= BMI between 25.0 to <30.0 kg/m^2^; and obese= BMI ≥30 kg/m^2^).

### Blood Sampling

Blood samples (5 ml) in EDTA tubes were drawn 10–14 days after receiving the first and second doses of either vaccine.

### Enzyme-Linked Immunosorbent Assay

The enzyme-linked immunosorbent assay (ELISA) was implemented according to a previously published method (16). Briefly, a 96-well plate was coated with the SARS-CoV-2 spike protein (S) antigen and incubated at 4°C overnight. Plates were washed 5 times with a washing solution. Diluted serum samples (1:100) were then added to wells at 100 µL/well and incubated for 30 minutes at room temperature. After washing, 100 µL/well of alkaline phosphatase–conjugated IgG antibody was added for 30 minutes. Plates were washed 5 times, and 100 µL/well of p-NPP substrate (Sigma–Aldrich) was added and incubated in the dark for 30 minutes. The reaction was terminated by the addition of 100 µL/well of stopping solution (1.2 N sodium hydroxide, Reagecon, UK). Optical density (OD) was measured at 405 nm.

### Vaccine-Related Symptoms

Participants were asked about the symptoms they experienced after receiving the vaccine. Symptoms stated were headache, fever, pain injection site, and nausea. Participants were able to select multiple responses. A score of one was awarded to each participants who selected the symptom of a concern and then the total symptom score was calculated for each participants (maximum 4 points out of 4).

### Statistical Analysis

Data for categorical variables are presented as the frequency and proportion. Fisher’s exact test was used to compare proportions across different groups, and post-hoc testing was performed to further investigate significant associations. Bonferroni adjustment to alpha was used for post-hoc tests to correct for multiple testing. Data for continuous variables are presented as the mean ± standard deviation. The Shapiro–Wilk test was used to assess the distribution normality of the total symptom score. The Mann–Whitney U test and Kruskal–Wallis test were used to compare the means of vaccine-related symptoms between groups (e.g., Pfizer vs. AstraZeneca, men vs. women, and age groups). Logistic regression analysis was performed to investigate predictors of change in anti-S protein IgG antibody status after the first dose and after the second dose. All models were adjusted for participants’ age and sex. Variables were coded as follows: age (<35 years= 1, 35–44 years= 2, 45–54 years= 3, ≥55 years= 4); sex (male= 1, female= 2); health status (healthy= 0, immune-related diseases= 1, chronic diseases= 2); weight status for participants >18 years (underweight= 1, healthy weight= 2, overweight= 3, obese= 4); blood type (A group= 1, B group= 2, AB group= 3, O group= 4); previous SARS-CoV-2 infection (no= 0, yes= 1); vaccine type (Pfizer= 1, AstraZeneca= 2); change in results (no change= 0, change from negative to positive= 1). SPSS was used to analyze all data presented in this study (IBM Corp. Released 2011. IBM SPSS Statistics for Windows, Version 20.0. Armonk, NY: IBM Corp). All tests were two-tailed, and a 95% confidence level was used to assess the significance of the findings.

## Results

### Participants’ Characteristics

**Figure 1:**
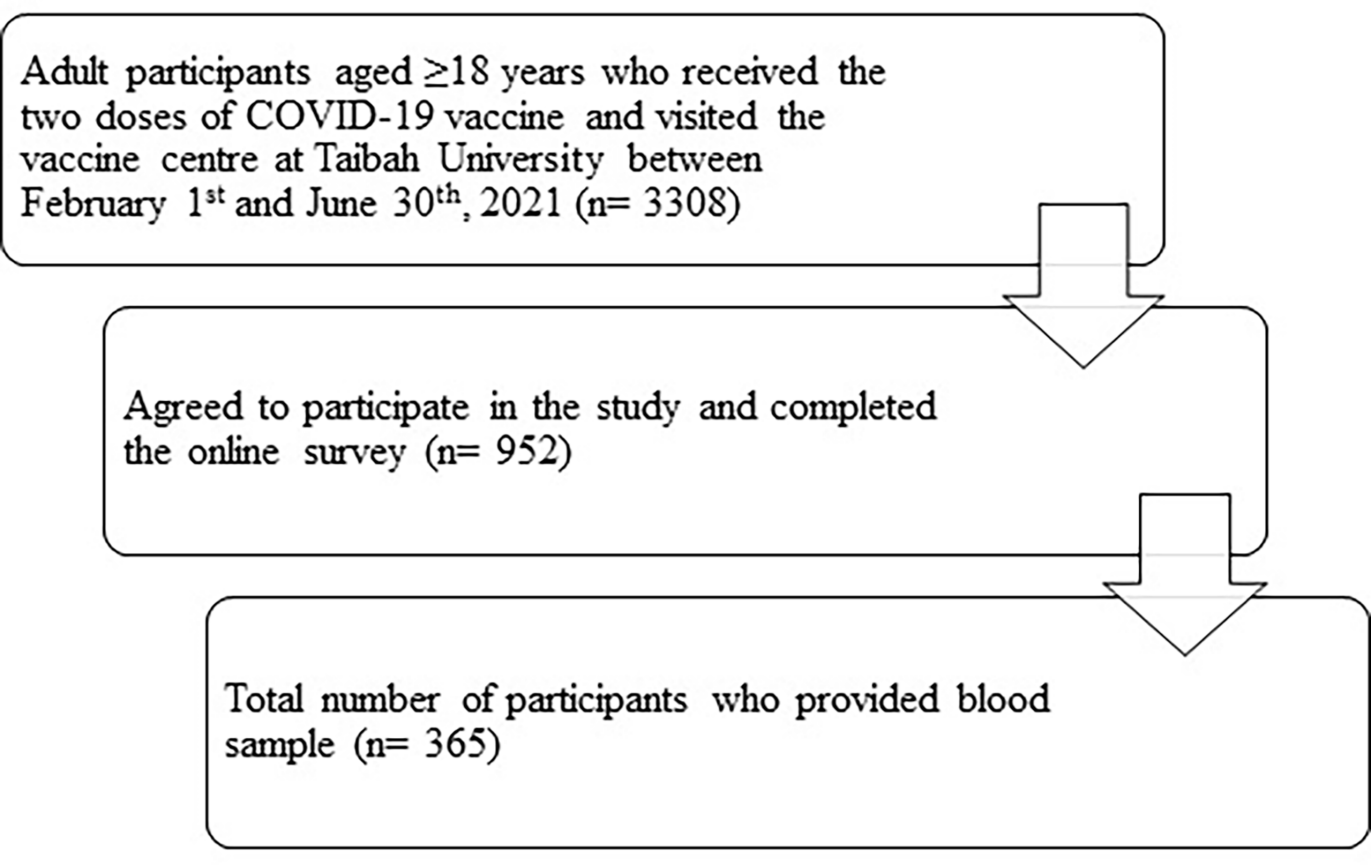
Flowchart of patient selection.

A total of 365 participants (38.3%) completed the study (**Figure 1**). One-quarter of the participants were 55 years or older (n= 90). The mean age of participants was 45.1 ± 14.7 years. Among the participants 84.0% (n = 306) were men and 81.6% (n = 298) were Saudis. Half of the participants held a bachelor’s or postgraduate degree (51.8%, n = 189), and 22.7% (n = 83) studied or worked in a health-related field. Immune-related diseases were reported by 8.2% of participants (n = 30). Healthy weight range was reported for 31.0% (n = 111) of participants, and 46.0% (n = 168) had type O blood. The majority of participants had not previously been diagnosed with SARS-CoV-2 infection (91.2%, n = 333). Half of the sample received the Pfizer vaccine (50.4%, n = 184). Detailed descriptions of the sociodemographic and health characteristics of the participants are presented in **Table 1**.

**Table 1:** Sociodemographic and health characteristics of participants (n = 365).

Variable	n	%
***Age***		
< 35 years	92	25.2
35-44 years	110	30.1
45-54 years	73	20.0
≥ 55 years	90	24.7
***Sex***		
Males	306	83.8
Females	59	16.2
***Nationality***		
Saudi	298	81.6
Non-Saudi	67	18.4
***Education level***		
< High-school	38	10.4
High-school/Diploma	138	37.8
Bachelor’s degree	138	37.8
Postgraduate	51	14.0
***Career***		
Health-related fields	83	22.7
Other fields	282	77.3
***Health status***		
Healthy	278	76.2
Immune-related diseases	30	8.20
Chronic diseases	57	15.6
Weight status^*^		
Underweight	28	7.73
Healthy weight	111	30.7
Overweight	98	27.1
Obese	125	34.5
***Blood type***		
A group	139	38.1
B group	49	13.4
AB group	9	2.50
O group	168	46.0
***Previous SARS CoV-2 infection***		
No	333	91.2
Yes	32	8.80
***Vaccine type***		
Pfizer	184	50.4
AstraZeneca	181	49.6

^*^Weight status was determined only for participants > 18 years (n = 362).

### Factors Related to Reactogenicity Profiles of COVID-19 Vaccines

We investigated the reactogenicity profiles after receiving the first dose of either the AstraZeneca or Pfizer vaccine, and 69.0% percent (n = 250) of participants reported experiencing at least 1 vaccine-related symptom. Pain at the injection site was the most frequently reported vaccine-related symptom (47.9%, n = 175), followed by fever (43.0%, n = 157) and headache (31.0%, n = 113); nausea was the least commonly reported symptom (2.20%, n = 8) and was only reported among those who received the AstraZeneca vaccine. Significantly higher proportions of participants who experienced headache, fever, pain at the injection site, and nausea received the AstraZeneca vaccine than received the Pfizer vaccine (90.3% vs. 9.70%, 85.4% vs. 14.6%, 60.6% vs. 39.4%, and 100% vs. 0.00%, respectively, *p* < 0.05). The associations between vaccine-related symptoms and vaccine type are shown in **Table 2**.

**Table 2:** Vaccine-related symptoms reported by participants stratified by type of vaccine.

Variable	Total (n= 365)	Pfizer (n= 184)	AstraZeneca (n= 181)	*p-value*
***Fever***				
No	208 (57.0)	161 (77.4)	47 (22.6)	< 0.001*
Yes	157 (43.0)	23 (14.6)	134 (85.4)	
***Headache***				
No	252 (69.0)	173 (68.7)	79 (31.3)	< 0.001*
Yes	113 (31.0)	11 (9.70)	102 (90.3)	
***Pain at the site of injection***				
No	190 (52.1)	115 (60.5)	75 (39.5)	< 0.001*
Yes	175 (47.9)	69 (39.4)	106 (60.6)	
***Nausea***				
No	357 (97.8)	184 (51.5)	173 (48.5)	0.003*
Yes	8 (2.20)	0 (0.00)	8 (100)	

Numbers presented in table are frequencies (percent).

*Statistically significant with 95% confidence level.

**Figure 2:**
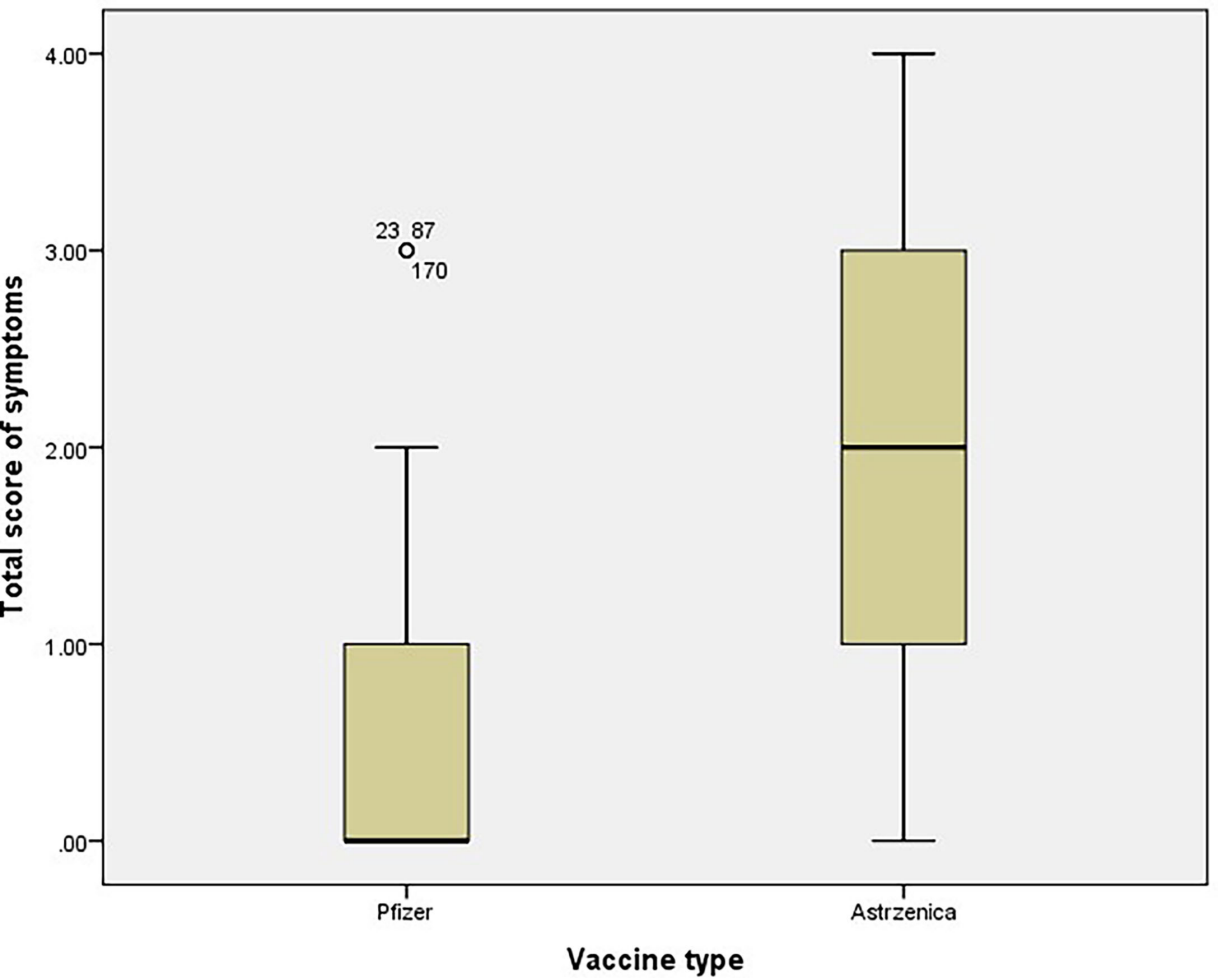
Total score of vaccine-related symptoms among participants who received Pfizer and AstraZeneca vaccines (n= 365).

**Figure 3:**
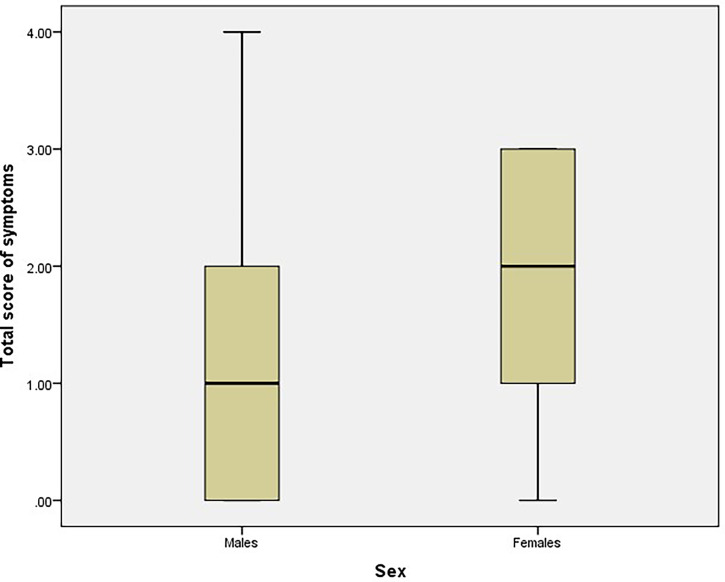
Total score of vaccine-related symptoms among males and females (n= 365).

**Figure 4:**
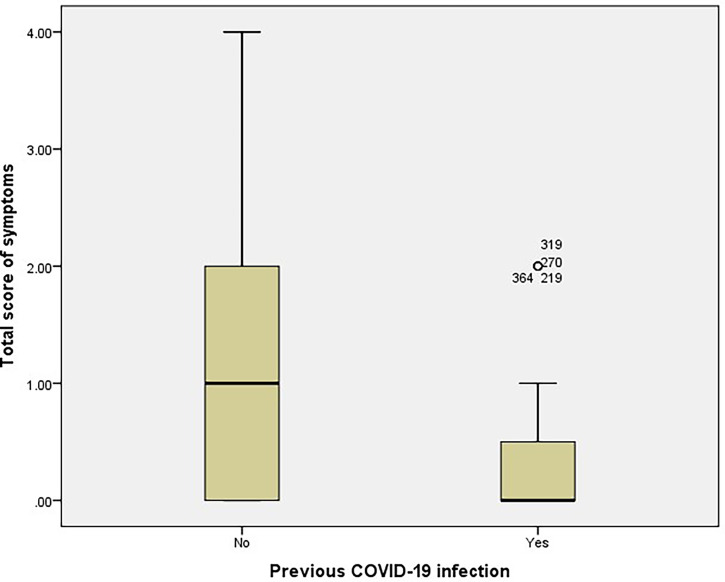
Total score of vaccine-related symptoms among participants with no previous infection and participants with reported previous COVID-19 infection (n= 365).

The total score of vaccine-related symptoms was calculated, and the mean total score was significantly higher among participants who received the AstraZeneca vaccine compared with those who received the Pfizer vaccine (1.93 ± 1.05 vs. 0.56 ± 0.67, respectively, *p* < 0.001, **Figure 2** **)**. Additionally, the mean total score for vaccine-related symptoms was significantly higher among women than among men (1.61 ± 1.17 vs. 1.17 ± 1.09, respectively, *p* = 0.006, **Figure 3** **)**. The mean total score of vaccine-related symptoms was also significantly higher among participants with no previous infection than among participants with reported any previous SARS-CoV-2 infection (1.32 ± 1.11 vs. 0.38 ± 0.71, respectively, *p* < 0.001, **Figure 4** **)**. The mean total scores of vaccine-related symptoms were similar across the different age groups (*p* = 0.914).

### Humoral Immune Response Following Receipt of Vaccines

To investigate the immunogenicity of the 2 vaccines, we used ELISA to measure the anti-S IgG antibody levels in blood samples collected from participants. The majority of participants developed a humoral immune response (83.3%, n = 304) after receiving the first dose of either vaccine, and most participants developed an anti-S IgG antibody response after receiving the second dose (98.9%, n = 361). We also found that 16% (n = 58) of participants did not develop an anti-S IgG antibody until after the second dose was received.

### Association Between Anti-S IgG Antibody Status and Sociodemographic Characteristics

Interestingly, we found that participants who developed an anti-S IgG antibody response after the first dose were significantly younger than those who did not develop any humoral immune response (44.3 ± 14.6 years vs. 48.7 ± 14.9 years, respectively, *p* = 0.030). A significant inverse relationship between age and anti-S IgG antibody status was found in this study (*p* = 0.032). We also performed *post hoc* testing, which showed that a significantly higher proportion of participants in the <35-year age group developed a humoral immune response after receiving the first dose of vaccines compared with the other age groups (*p* = 0.007). No association was found between age and anti-S IgG antibody status after receiving the second dose. Additionally, no associations were identified between other sociodemographic characteristics and anti-S IgG antibody status after receiving either vaccine dose.

### Association Between Anti-S IgG Antibody Status and Health Characteristics

Associations between anti-S IgG antibody status and health characteristics were observed after performing ELISA. We found that chronic disease was significantly associated with anti-S IgG antibody status after both the first and second doses (*p* < 0.01 for both time points). Additionally, *post hoc* testing showed that a significantly higher proportion of healthy participants developed humoral immune responses after the first dose compared with participants with immune-related diseases and those with chronic diseases (*p* = 0.002 and *p* = 0.003, respectively). The data showed that the proportions of participants stratified by anti-S IgG antibody status were similar across all weight groups after the first dose, whereas after receiving the second dose, a significantly lower proportion of underweight participants developed a humoral immune response compared with participants classified as healthy weight, overweight, or obese (*p* = 0.001). Associations between participants’ health characteristics and anti-S IgG antibody status are presented in **Table 3**.

**Table 3:** Associations between participants’ characteristics and anti-S IgG antibody status (n = 365).

Variable	First dose		*p-value*	Second dose		*p-value*
	Negative (n = 61)	Positive (n = 304)		Negative (n = 4)	Positive (n = 361)	
**Age, n (%)**						
< 35 years	7 (7.60)	85 (92.4)	0.032*	0 (0.00)	92 (100)	0.672
35-44 years	20 (18.2)	90 (81.8)		2 (1.80)	108 (98.2)
45-54 years	14 (19.2)	59 (80.8)		1 (1.40)	72 (98.6)
≥ 55 years	20 (22.2)	70 (77.8)		1 (1.10)	89 (98.9)
**Sex, n (%)**						
Male	52 (17.0)	254 (83.0)	0.850	4 (1.30)	302 (98.7)	1.00
Female	9 (15.3)	50 (84.7)		0 (0.00)	59 (100)
**Nationality, n (%)**						
Saudi	51 (17.1)	247 (82.9)	0.856	4 (1.30)	294 (98.7)	1.00
Non-Saudi	10 (14.9)	57 (85.1)		0 (0.00)	67 (100)
**Education level, n (%)**						
< High-school	7 (18.4)	31 (81.6)	0.250	0 (0.00)	38 (100)	0.877
High-school/Diploma	17 (12.3)	121 (87.7)		1 (0.70)	137 (99.3)
Bachelor’s degree	25 (18.1)	113 (81.9)		2 (1.40)	136 (98.6)
Postgraduate	12 (23.5)	39 (76.5)		1 (2.00)	50 (98.0)
**Career, n (%)**						
Health-related fields	10 (12.0)	73 (88.0)	0.242	0 (0.00)	83 (100)	0.578
Other fields	51 (18.1)	231 (81.9)		4 (1.40)	278 (98.6)
**Health status, n (%)**						
Healthy	37 (13.3)	241 (86.7)	<0.001*	0 (0.00)	278 (100)	0.001*
Immune-related diseases	2 (6.70)	28 (93.3)		0 (0.00)	30 (100)
Chronic diseases	22 (38.6)	35 (61.4)		4 (7.00)	53 (93.0)
**Weight status ^1^, n (%)**						
Underweight	7 (25.0)	21 (75.0)	0.584	2 (7.10)	26 (92.9)	0.007*
Healthy weight	20 (18.0)	91 (82.0)		2 (1.80)	109 (98.2)
Overweight	15 (15.3)	83 (84.7)		0 (0.00)	98 (100)
Obese	19 (15.2)	106 (84.8)		0 (0.00)	125 (100)
**Blood type, n (%)**						
A group	20 (14.4)	119 (85.6)	0.655	2 (1.40)	137 (98.6)	1.00
B group	7 (14.3)	42 (85.7)		0 (0.00)	49 (100)
AB group	1 (11.1)	8 (88.9)		0 (0.00)	9 (100)
O group	33 (19.6)	135 (80.4)		2 (1.20)	166 (98.8)
**Previous SARS CoV-2 infection, n (%)**						
No	58 (17.4)	275 (82.6)	0.325	4 (1.20)	329 (98.8)	1.00
Yes	3 (9.40)	29 (90.6)		0 (0.00)	32 (100)
**Direct exposure with infected case, n (%)**						
No	49 (18.4)	218 (81.6)	0.206	4 (1.50)	263 (98.5)	0.577
Yes	12 (12.2)	86 (87.8)		0 (0.00)	98 (100)
**Vaccine type, n (%)**						
Pfizer	24 (13.0)	160 (87.0)	0.068	1 (0.50)	183 (99.5)	0.369
AstraZeneca	37 (20.4)	144 (79.6)		3 (1.70)	178 (98.3)

*Statistically significant with 95% confidence level.

^1^Weight status was determined only for participants > 18 years (n = 362).

### Predictors of Change in Anti-S IgG Antibody Status

Logistic regression analysis showed that no health characteristics were able to significantly predict the seropositivity in anti-S IgG antibody after either the first dose or the second dose. Only the presence of chronic diseases predicted higher odds of a seropositivity in anti-S IgG antibody status compared with healthy participants with no chronic diseases (adjusted odds ratio [aOR]: 2.95, 95% confidence interval: 1.44–5.46, *p* = 0.002, **Table 4** **)**.

**Table 4:** Predictors of change in the anti-S IgG antibody status after the first and second dose (n = 365).

	aOR	95% Confidence Interval	*p-value*
***Health status***			
Healthy	*Reference Category*		
Immune-related diseases	0.44	0.10 to 1.93	0.275
Chronic diseases	2.95	1.44 to 5.46	0.002*
***Weight status* ^1^**			
Underweight	*Reference Category*		
Healthy weight	0.97	0.32 to 2.92	0.959
Overweight	0.86	0.28 to 2.65	0.795
Obese	0.89	0.30 to 2.68	0.838
***Blood type***			
A group	*Reference Category*		
B group	1.01	0.38 to 2.69	0.985
AB group	0.65	0.08 to 5.59	0.695
O group	1.48	0.78 to 2.80	0.231
***Previous SARS CoV-2 infection***			
No	*Reference Category*		
Yes	0.48	0.14 to 1.66	0.244
***Direct exposure with infected case***			
No	*Reference Category*		
Yes	0.71	0.35 to 1.43	0.334
***Vaccine type***			
Pfizer	*Reference Category*		
AstraZeneca	1.76	0.98 to 3.14	0.058

All models were adjusted for participants’ age and sex.

^1^Weight status was determined only for participants > 18 years (n = 362).

*Statistically significant with 95% confidence level.

## Discussion

Most participants reported at least 1 vaccine-related symptom. Pain at the injection site was the most frequently reported vaccine-related symptom. Significantly higher proportions of individuals who experienced headache, fever, pain at the injection site, and nausea received the AstraZeneca vaccine than received the Pfizer vaccine. The mean total vaccine-related symptom score was significantly higher among participants who received the AstraZeneca vaccine, women, and participants with no previous history of COVID-19 infection. Anti-S IgG antibodies were detected in 98.9% of participants after receiving both vaccine doses, including 99.5% of participants who received the Pfizer vaccine and 98.3% of participants who received the AstraZeneca vaccine. A significantly higher proportion of participants in the <35-year age group developed a humoral immune response after the first vaccine dose compared with participants in other age groups. Lower proportions of underweight participants and those with chronic diseases developed a humoral immune response following receiving both vaccine doses.

COVID-19 vaccines have been used with huge success in several countries globally; however, greater than half of the worldwide population remains unvaccinated (44.7% of the world population has received at least one dose of any COVID-19 vaccine; https://ourworldindata.org/covid-vaccinations, accessed on 28 September 2021).

Investigations examining associations between vaccination side effects and immunogenicity are limited and remain necessary to assess vaccine efficacy. Adverse reactions that occur following vaccination are recognized as being predictive indicators of a virtuous immune response; however, inadequate evidence regarding the reactogenicity of the COVID-19 vaccines has been published (17). In the current study, the data showed that 70% of participants described at least 1 vaccine-related symptom, with pain at the injection site being the most commonly reported vaccine-related symptom, followed by fever, headache, and nausea. No participants reported needing medical attention or hospitalization after receiving their first dose. In addition, the data showed that recipients of the AstraZeneca vaccine reported more reactogenicity events following vaccination than those who received the Pfizer vaccine. We showed that women reported experiencing more vaccine-related symptoms than men. The findings of this study are in line with the findings reported in the UK, in which high reactogenicity was identified among women compared with men following vaccination (18).

Most participants included in the current study reported no previous SARS-CoV-2 infection; therefore, they were primed with the first vaccine dose and reported significantly higher vaccine-related adverse events than those with previous SARS-CoV-2 infection. The results of this study are consistent with those of a previous study, which showed that reactogenicity was more commonly reported by infection-naive individuals, whereas fewer reactogenicity responses were reported among participants with prior COVID-19 infection (19). However, inconsistent findings were reported by another study, which showed that participants with confirmed SARS-CoV-2 infections reported more reactogenicity symptoms after receiving the first dose of both types of vaccine (Pfizer and AstraZeneca) (18). This inconsistent finding may be due to differences between the methods used to assess vaccine reactogenicity. Genetic variations among the studied populations (Middle Eastern vs. Caucasian) may also contribute to discrepancies. Additionally, the bias of respondents was likely to influence the incidence and degree of side effects reported by participants. Last, variations among individual pain tolerances could introduce bias in the reporting of vaccine-related symptoms.

Immunogenicity is a fundamental feature of vaccine development. Data from clinical trials have revealed that immunogenicity is the mechanism underlying vaccine efficiency. Evaluating circulating antibody intensities allows for the evaluation of diverse vaccinated populations, although understanding the contributors to variations remains challenging, and no predictors for determining the protective efficacy of COVID-19 vaccines have been identified thus far (20).

In our study, we explored the immunogenicity of 2 vaccines and investigated the vaccine-induced humoral immune response. We showed that 83.3% of the participants developed a humoral immune response after receiving the first dose of either vaccine. Almost all participants (98.9%) developed a humoral immune response after receiving the second dose, indicating that 16% of the total population developed an immune response only after the second dose. Therefore, a second dose was necessary to achieve a higher seroconversion percentage. A recent study showed that approximately 20% of hospitalized patients received at least one vaccine dose, although several patients had not received a second dose (21).

Remarkably, our study showed that younger participants in the <35-year age group experienced a significantly increased seropositivity rate (92.4%) and generated anti-S IgG antibodies after the first dose compared with participants in other age groups (35–44, 45–54, and ≥55 years). The second COVID-19 vaccine dose is necessary to increase antibody levels for protection from the infection. Our results are in agreement with the results of several studies that have reported an age-dependent immune response to COVID-19 vaccination, which concluded that anti-S antibody levels were significantly lower among older vaccine recipients (22, 23).

Our study showed that the presence of chronic diseases (e.g., type 2 diabetes, hypertension, and cardiovascular disease) was associated with lower seropositivity rates. Healthy participants were expected to develop a humoral immune response regardless of the number of doses received compared with participants with immune-related diseases or chronic diseases. However, a higher proportion of vaccine recipients with immune-related diseases developed a humoral immune response than vaccine recipients with chronic diseases. SARS-CoV-2 vaccination in healthy people has been reported to produce immune protection against COVID-19 in both immune system arms, humoral and cellular immune responses (24).

Among participants who developed humoral immune responses, the proportion of underweight participants was significantly lower than those of other weight groups (healthy weight, overweight, and obese). In fact, the prevalence of overweight and obesity in Saudi Arabia is very high. In 2019, the prevalence of overweight and obesity among Saudi adults in Jeddah City was 35.1% and 34.8%, respectively (25). In addition, nationally representative data collected in 2020 show high prevalence of obesity among Saudis of 24.7% (26). Thus, findings of our study can be generalizable to the general population of adults in Saudi Arabia.

Underweight individuals are commonly malnourished and more likely to present with inadequate levels of multiple nutrients that play key roles in the function of the immune system. Population-level malnutrition has been associated with increased proportions of fatal COVID-19 in regions with higher undernutrition burdens (27). Thus, interventions that aim to correct for any nutritional deficiencies might be worthy of exploration in future studies to maximize the efficacy of COVID-19 vaccinations programs.

Our study is the first to compare the reactogenicity and immunogenicity of the most commonly used vaccines, Pfizer and AstraZeneca, in Saudi Arabia and several other settings. However, this study is limited by its cross-sectional design, preventing the long-term effects of vaccination from being evaluated. Furthermore, data concerning ethnicity were lacking; thus, we were unable to control for this variable in our analysis. However, data concerning ethnicity were not collected, as previous research in Saudi Arabia showed no association between ethnicity and immunogenicity (unpublished data). Information concerning health conditions, previous COVID-19 infection, and side effects following vaccination were self-reported, which could introduce temporal gaps in the reporting. In addition, we did not assess the associations of cellular immune responses with suitable reactogenicity profiles. We emphasize that our investigation in the current work is observational rather than serving as a formal evaluation.

## Conclusion

Findings of this study suggest that different levels of reactogenicity and immunogenicity may be observed depending on the vaccine type, age, chronic disease status, and weight status. Participants who received the AstraZeneca vaccine reported more vaccine-related symptoms than those who received the Pfizer vaccine; however, both vaccines were well-tolerated and effective. These data could assist governments and policy-makers in the establishment of regulations concerning the provision of paid leave to promote vaccination, in addition to guiding individuals in scheduling their commitments post-vaccination. Future research should explore the long-term health outcomes related to commonly used vaccines at the national and international levels. Additionally, future studies should investigate the impacts of nutritional status on the efficacy of the COVID-19 vaccinations.

## Data Availability Statement

The raw data supporting the conclusion of this article will be made available by the authors, without undue reservation.

## Ethics Statement

The studies involving human participants were reviewed and approved by the College of Applied Medical Sciences, Taibah University (2021/96/117/MLT). The patients/participants provided their written informed consent to participate in this study.

## Author Contributions

WMa and WMu contributed to conception and design of the study. WMa and WMu organized the database. WMu performed the statistical analysis. WMa wrote the first draft of the manuscript. WMu wrote sections of the manuscript. All authors contributed to manuscript revision, read, and approved the submitted version.

## Funding

The authors extend their appreciation to, Taibah University, represented by the Deanship of Scientific Research, for funding this project# RC-442/4.

## Conflict of Interest

The authors declare that the research was conducted in the absence of any commercial or financial relationships that could be construed as a potential conflict of interest.

## Publisher’s Note

All claims expressed in this article are solely those of the authors and do not necessarily represent those of their affiliated organizations, or those of the publisher, the editors and the reviewers. Any product that may be evaluated in this article, or claim that may be made by its manufacturer, is not guaranteed or endorsed by the publisher.
